# Electrodes Based on Carbon Aerogels Partially Graphitized by Doping with Transition Metals for Oxygen Reduction Reaction

**DOI:** 10.3390/nano8040266

**Published:** 2018-04-23

**Authors:** Abdalla Abdelwahab, Jesica Castelo-Quibén, José F. Vivo-Vilches, María Pérez-Cadenas, Francisco J. Maldonado-Hódar, Francisco Carrasco-Marín, Agustín F. Pérez-Cadenas

**Affiliations:** 1 Carbon Materials Research Group, Department of Inorganic Chemistry, Faculty of Sciences, University of Granada, Campus Fuentenueva s/n, ES18071-Granada, Spain; abdalla.abdelwahab85@gmail.com (A.A.); jesicacastelo@ugr.es (J.C.-Q.); joseviv@ugr.es (J.F.V.-V.); fmaldon@ugr.es (F.J.M.-H.); fmarin@ugr.es (F.C.-M.); 2 Department of Inorganic and Technical Chemistry, Science Faculty, UNED, Paseo Senda del Rey 9, ES28040-Madrid, Spain; mariaperez@ccia.uned.es

**Keywords:** carbon aerogel, graphitic cluster, metal nanoparticle, oxygen reduction reaction, electro-catalysis

## Abstract

A series of carbon aerogels doped with iron, cobalt and nickel have been prepared. Metal nanoparticles very well dispersed into the carbon matrix catalyze the formation of graphitic clusters around them. Samples with different Ni content are obtained to test the influence of the metal loading. All aerogels have been characterized to analyze their textural properties, surface chemistry and crystal structures. These metal-doped aerogels have a very well-developed porosity, making their mesoporosity remarkable. Ni-doped aerogels are the ones with the largest surface area and the smallest graphitization. They also present larger mesopore volumes than Co- and Fe-doped aerogels. These materials are tested as electro-catalysts for the oxygen reduction reaction. Results show a clear and strong influence of the carbonaceous structure on the whole electro-catalytic behavior of the aerogels. Regarding the type of metal doping, aerogel doped with Co is the most active one, followed by Ni- and Fe-doped aerogels, respectively. As the Ni content is larger, the kinetic current densities increase. Comparatively, among the different doping metals, the results obtained with Ni are especially remarkable.

## 1. Introduction

Nowadays the development of electric vehicles is one of the most promising alternatives to replace combustion engines and therefore there is a high interest in finding new environmental friendly sources of energy for automotive applications. For this reason, the production of electrical energy from chemical reactions by using fuel cells is a really interesting matter from both the industrial and fundamental research points of view [1,2,3]. Oxygen Reduction Reaction (ORR) takes place on the cathode in a fuel cell and several works can be found in the published literature about the synthesis and optimization of electro-catalytic materials for this reaction [1,2,3,4,5,6,7,8,9,10,11]. Of these, platinum-based electro-catalysts happen to be the most widely studied, since Pt is the most active metal for ORR [1,6,7,8,9,10]. Nevertheless, the rising price of platinum and other precious metals as Pd or Ir makes it more difficult to commercialize devices containing them. This is the reason why non precious metal electro-catalysts are more numerous and more studied in order to lower the costs of fuel cells [2,5,6,7,8,9,10,11,12].

On the other hand, carbon-based materials are being seriously considered as optimal candidates for ORR electro-catalysts [2,11,13,14]. Carbon gels are nanostructured materials, they are obtained from organic gels after their carbonization. Organic gels are prepared by polycondensation of organic monomers, normally resorcinol (R) and formaldehyde (F) [15]. The textural characteristics of carbon gels strongly depend on a precise control of the reactant concentrations and the conditions of the synthesis process: gelation, curing, drying and carbonization [16,17,18]. Surfactants can be added during the R-F polymerization which influences the morphology of the doped carbon gels and the metal dispersion [19]. Both surface area and pore volume, including the pore size distribution, are properties related to the synthesis conditions and processing that can be tuned, enabling the development of a wide set of materials with remarkable properties, e.g., for adsorption [20,21], catalysis [22,23,24,25] and electrochemical applications [26,27]. Besides, carbon gels doped with transition metals exhibit a homogeneous distribution together with a high dispersion of the metals throughout the carbon matrix [28,29]. Thus, transition metals are used in order to be anchored into the carbon matrix, which minimizes their leaching in liquid phase applications [19].

In the present work, carbon aerogels doped with iron, cobalt, and nickel were prepared, including a different nickel loading, exhaustibly characterized from textural, chemical, and electro-chemical points of view. Finally, their performances as electro-catalysts for the oxygen reduction reaction were evaluated and discussed in terms of their differences in porous texture, chemical characteristics, and metal doping, being the results of this comparative study the main objective of this work.

## 2. Materials and Methods

### 2.1. Preparation and Characterization of the Materials

Carbon aerogels doped with Ni, Co and Fe were prepared from resorcinol (R) and formaldehyde (F) dissolved in water (W) and using nickel, cobalt, or iron acetate as a catalyst precursor (C). The molar ratios were R:F = 1:2 and R:W = 1:17. Fe- and Co-doped aerogels were prepared only with a 6 wt %, approx. of metal loading while Ni-doped aerogels were prepared with 1, 4 and 6 wt %, approx. varying the amount of C. When an organic sol-gel solution was obtained it was cast into glass molds. After that, the curing process to obtain the organic gels was: 1 day at 40 °C, and 5 days at 80 °C. The glass molds were broken, and the organic aerogels were immersed in acetone for 24 h. Finally, the organic aerogels were treated with supercritical CO_2_ for their drying. Another sample (A0), to be used as reference, was also prepared but without any transition metal. The organic aerogels were carbonized at 900 °C to obtain the carbon gels using a N_2_ flow and a heating rate of 1 °C min^−1^. The obtained carbon aerogels (A) were named as: ANi1, ANi4, ANi6, AFe6 and ACo6, the numbers indicate the approximate metal content in percentage. The metal loadings of the aerogels were determined by burning off a portion of a sample at 900 °C in air and weighting the residue.

The aerogels were texturally characterized by gas adsorption, scanning electron microscopy (SEM), and high-resolution transmission electron microscopy (HRTEM), and chemically characterized by X-ray diffraction (XRD), Raman spectroscopy and X-ray photoelectron spectroscopy (XPS). Samples performance for Oxygen Reduction Reaction was tested by means of cyclic voltammetry (CV) and linear sweep voltammetry (LSV).

N_2_ and CO_2_ adsorptions were carried out at −196 °C and 0 °C, respectively. Prior to measuring, the samples were outgassed for 24 h at 110 °C under high vacuum (10^−6^ mbar). The BET equation was applied to the N_2_ adsorption obtaining the apparent surface area, *S*_BET_. The Dubinin-Radushkevich (DR) equation was applied to the N_2_ and CO_2_ adsorption data and the corresponding micropore volume (*W*_0_) and micropore mean width (*L*_0_) were obtained. Total pore volumes (*V*_0.95_) were determined from the N_2_ adsorption isotherms at −196 °C and at 0.95 relative pressure. Finally, the mesopore volumes (*V*_BJH_) and the mean mesopore widths (*L*_BJH_) were obtained applying the BJH method [30] to the desorption branch of the N_2_ isotherms.

SEM was carried out using a Zeiss SUPRA40VP scanning electron microscope (Carl Zeiss AG, Oberkochen, Germany), equipped with a secondary electron detector, back-scatter electron detector, and using a X-Max 50 mm energy dispersive X-ray microanalysis system. All the samples were crushed before realizing this analysis.

HRTEM was performed using a FEI Titan G2 60-300 microscope (FEI, Eindhoven, The Netherlands) with a high brightness electron gun (X-FEG) operated at 300 kV and equipped with a Cs image corrector (CEOS) and for analytical electron microscopy (AEM) a SUPER-X silicon-drift window-less EDX detector. The AEM spectra were collected in STEM (scanning transmission electron microscopy) mode using a HAADF (high angle annular dark field) detector. Digital X-ray maps were also collected on selected areas of the samples.

Raman spectra were recorded using a Micro-Raman JASCO NRS-5100 dispersive spectrometer (JASCO Inc, Easton, MD, USA) with a 532 nm laser line. From these spectra the ratio *I*_G_/*I*_D_ was calculated as the quotient between the maximum intensity of each band.

XRD analysis was carried out with BRUKER D8 ADVANCE diffractometer (BRUKER, Rivas-Vaciamadrid, Spain) using CuK radiation. JCPDS files were used to assign the different diffraction peaks observed. Diffraction patterns were recorded between 10° and 70° (2θ) with a step of 0.02° and a time per step of 96 s. The average crystal size (*d*_XRD_) was determined using the Scherrer equation.

XPS measurements of the carbon aerogels were performed using a Physical Electronics ESCA 5701 (PHI, Chanhassen, MN, USA) equipped with a MgK X-ray source (*hν* = 1253.6 eV) operating at 12 kV and 10 mA, and a hemispherical electron analyzer. The obtained binding energy (BE) values were referred to the C_1s_ peak at 284.7 eV. A base pressure of 10^−9^ mbar was maintained during data acquisition. Survey and multi-region spectra were recorded at C_1s_, O_1s_, Fe_2p_, Co_2p_ and Ni_2p_ photoelectron peaks. Each spectral region was scanned enough times to obtain adequate signal-to-noise ratios. The spectra obtained after a background signal correction were fitted to Lorentzian and Gaussian curves to obtain the number of components, the position of each peak and the peak areas.

### 2.2. Electro-Chemical Studies. Oxygen Reduction Reaction

Cyclic Voltammetry (CV) and Linear Sweep Voltammetry (LSV) experiments were conducted on a three-electrode cell controlled by a Biologic VMP multichannel potentiostat (Bio-Logic Spain, Barcelona, Spain). A Rotating Disk Electrode (RDE) Metrohm AUTOLAB RDE-2 with a 3 mm Glassy Carbon tip (Gomensoro S.A, Madrid, Spain) was used as a working electrode. 5 mg of electro-catalyst were suspended on 1 mL of a solution which contained Nafion (5%) and water in a 1:9 (*v*:*v*) ratio. Subsequently, 10 µL of this suspension were loaded on RDE tip and dried under an infrared lamp [14]. The glassy carbon electrode had been previously polished with 1, 0.3 and 0.05 µm alumina powder and sonicated in deionized water and ethanol. Ag/AgCl was chosen as a reference electrode and Pt-wire as a counter electrode. The three electrodes were immersed in a 0.1 M KOH (electrolyte) solution in water.

The oxygen reduction reaction may occur by two different pathways: one implies 2 $e_{s}^{-}$ transference and the formation of peroxide species (Equation (1)) which could damage the electro-catalytic layer which is not desirable; the other leads only to the formation of hydroxide and it occurs by a 4 $e_{s}^{-}$ (Equation (2)) which is the requested one.

(1) $$O_{2}+H_{2}O+2e_{s}^{-}\;\to {\;\mathrm{HO}}_{2}^{-}\;+{\mathrm{OH}}^{-}$$

(2) $$O_{2}+2{\;H}_{2}O+4e_{s}^{-}\;\to \;4{\;\mathrm{OH}}^{-}$$

CV experiments were carried out while N_2_ or O_2_ bubbled through the electrolyte solution during the measurements. The chosen potential window ranged from −0.8 to 0.4 V (at 5 mV·s^−1^ and 50 mV·s^−1^). LSV curves were obtained in O_2_-saturated 0.1 M KOH solutions at a different rotation speed and sweeping voltage, from 0.4 to −0.8 V (5 mV·s^−1^). Data were fitted to the Koutecky-Levich model (Equations (3) and (4)) in order to evaluate the electro-catalytic performance of the samples and the transferred electron number for each of them [14].

(3) $$\frac{1}{j}=\frac{1}{j_{k}}+\frac{1}{B\omega^{0.5}}$$

(4) $$B=0.2nF{\left(D_{O_{2}}\right)}^{2/3}v^{-1/6}C_{O_{2}}$$

where *j*, current density; *j_k_*, kinetic current density; *ω*, rotation speed; *F*, Faraday constant; $D_{O_{2}}$, oxygen diffusion coefficient (1.9 × 10^−5^ cm^2^·s^−1^); *ν*, viscosity (0.01 cm^2^·s^−1^); $C_{O_{2}}$, oxygen concentration (1.2 × 10^−6^ mol·cm^−3^).

## 3. Results

Table 1 collects the names and the textural properties of the samples. All carbon aerogels are microporous and mesoporous materials with significant mesopore volumes and BET surfaces areas. Aerogels doped with Ni have the highest surface areas and pore volumes among the metal-doped samples, specially the highest micropore volumes; among the Ni samples ANi6 is the most microporous material. In the opposite site, AFe6 has the lowest surface area and pore volumes. Aerogel A has textural properties comparable with the rest of samples.

**Figure 1:**
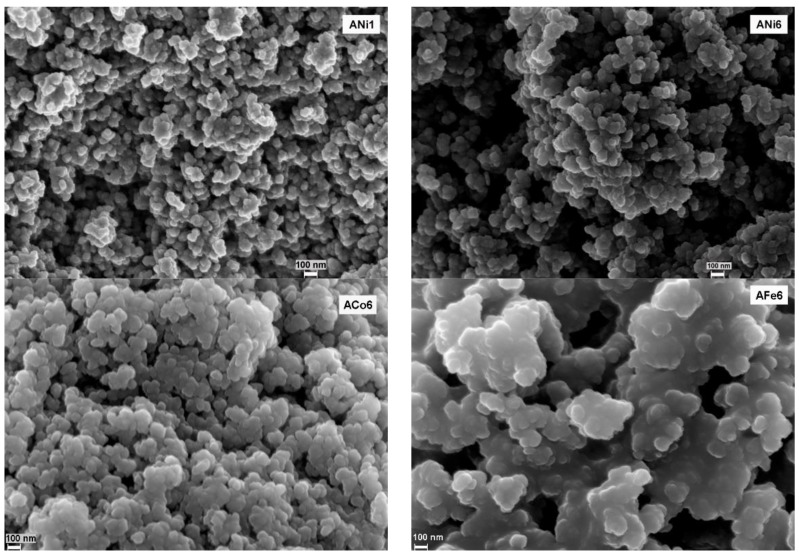
SEM microphotographs obtained at 100.00 KX of magnification of the samples ANi1, ANi6, ACo6 and AFe6.

Figure 1 shows the morphology of the samples studied by SEM. The structure of the carbon gels consist in a network formed by rounded particles with a different degree of fusion [31]; a very well-developed macroporous structure is also observed. No significant morphological differences are observed by SEM among the samples.

**Figure 2:**
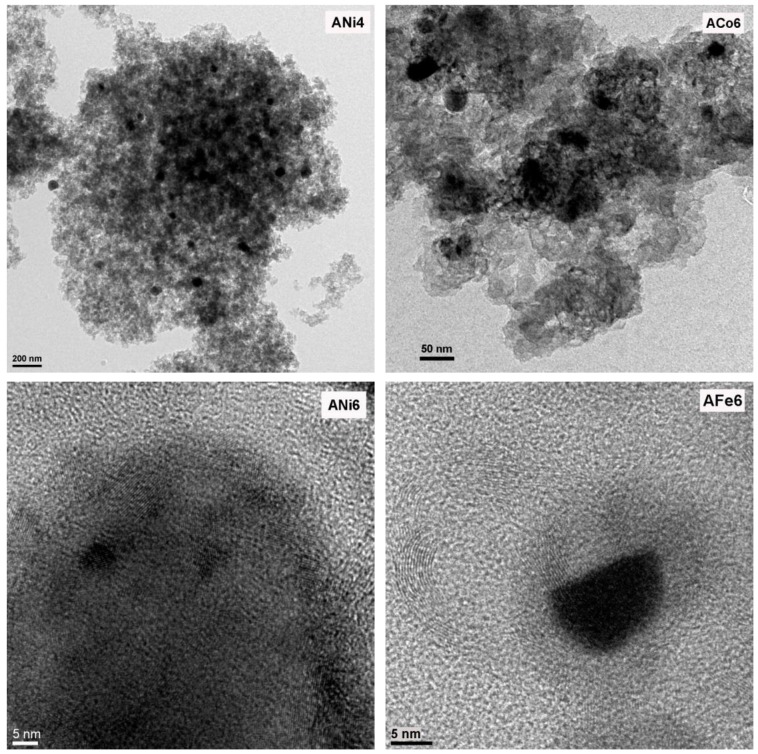
HRTEM images of the samples ANi4, ACo6, ANi6 and AFe6.

**Figure 3:**
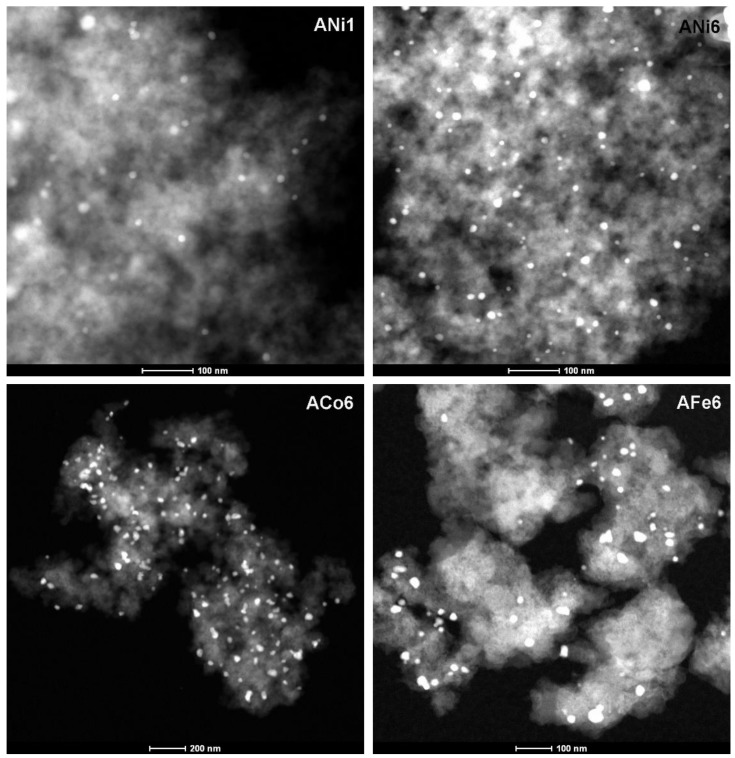
AEM spectra collected in STEM mode using a HAADF detector of the samples ANi1, ACo6, ANi6 and AFe6.

**Figure 4:**
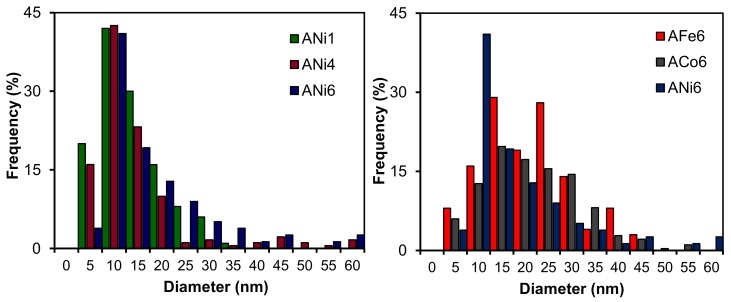
Particle size distributions obtained from HRTEM images.

**Figure 5:**
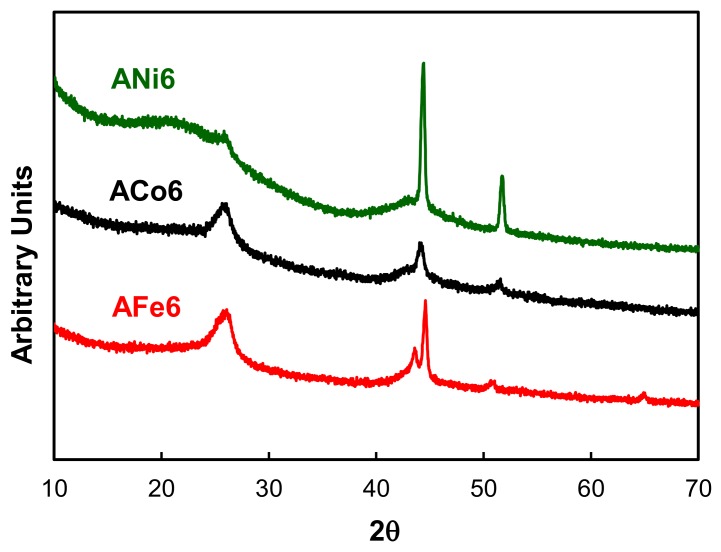
XRD patterns of aerogels ANi6, ACo6 and AFe6.

Regarding the metal phase characterization, HRTEM analysis indicate that metals are mainly embedded within the carbon matrix; metal particles are clearly shown in Figure 2 and Figure 3, and these are very well dispersed throughout the aerogel texture. Moreover, metal particles are detected within a wide range of nanometric sizes (Figure 4). On the other hand, these metal nanoparticles have catalyzed a partial graphitization around them during the pyrolysis; this fact was observed in all cases. Good examples of these graphitic clusters are shown in Figure 2, aerogels ANi6 and AFe6. It should be noted that the above-mentioned graphitization was not observed in sample A by HRTEM. These graphite clusters in the doped carbon aerogel structure were also observed in other works [31,32] with Co, Fe and Ni as doping metals. This can also be detected by XRD as a wide signal at around 26 θ (peak 002 of graphite, JCPDS card No. 41-1487) specially in the case of aerogels ACo6 and AFe6 (Figure 5), although this signal hardly can be observed in the Ni doped samples. This would indicate that the graphitic clusters in the Ni samples probably have mean crystallite sizes smaller than 4 nm or a very thin laminar form.

On the other hand, the XRD peaks in Figure 5 clearly show the presence of Ni and Co completely reduced (JCPDS cards No. 04-0850, and 15-0806, respectively). Only in the case of sample AFe6 a mixture of Fe (0) (peaks at 44.6° and 65.1°, (JCPDS card No. 06-0696)) and Fe (III) (at 43.5°) could be detected; although the coincidence of this signal with the peak (101) of graphite makes both its assignation and resolution, difficult.

**Figure 6:**
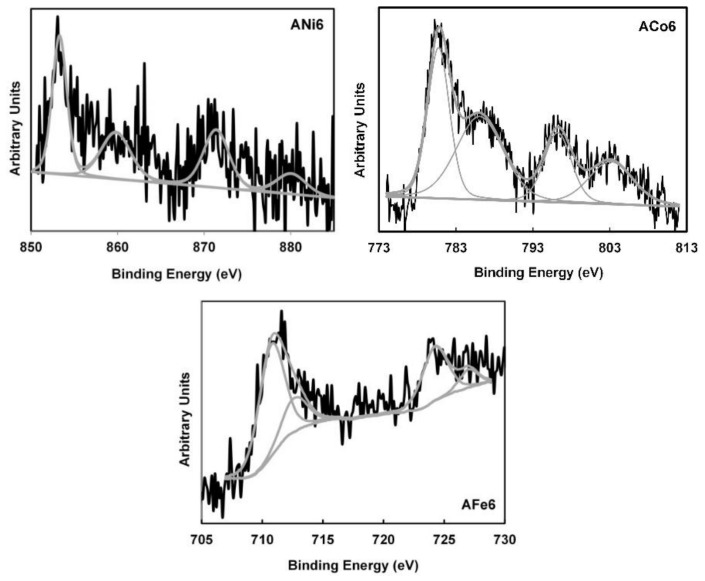
XP spectra of the doped carbon aerogels.

Analyzing the XP spectra, the peaks corresponding with metal phases cannot be practically distinguished from the base line in the case of ANi1 and ANi4, this means that Ni concentration on the external surface of these samples can be considered negligible. Only Ni2p, Co2p and Fe2p spectra of aerogels with 6 wt % could be analyzed. Figure 6 shows in the Ni2p spectrum only one Ni2p_3/2_ signal at 853.3 eV which is assigned to Ni (II) [33]; its corresponding satellite peak can be clearly observed at 859.8 eV. In this line, only one Co2p_3/2_ signal is observed at 781.1 eV of BE together its corresponding satellite at 786.1 eV, which is also assigned to Co (II) species [33]. Finally, the Fe2p spectrum contains two species of iron at 710.7 and 712.7 eV being these signals assigned to Fe_2_O_3_ (76.5%) and Fe_3_O_4_ (23.5%), respectively [33].

**Figure 7:**
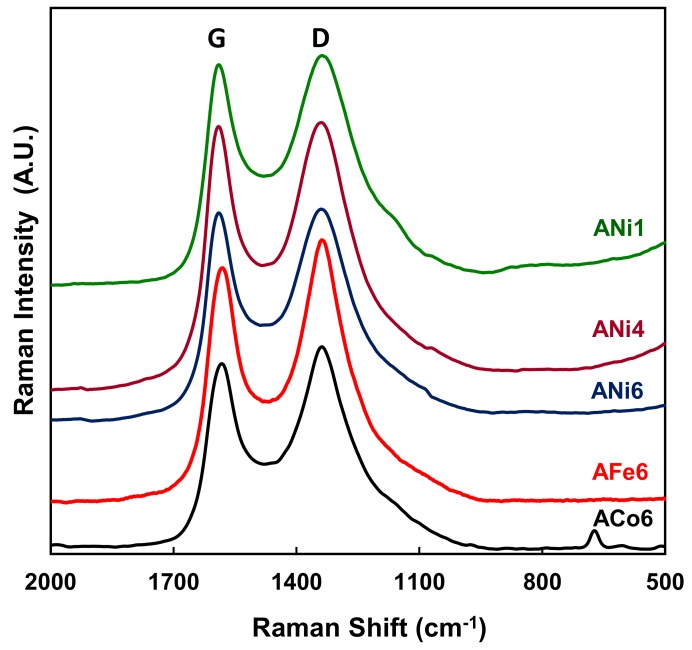
Raman spectra of the doped carbon aerogels.

Raman spectra show (Figure 7) two main peaks at 1340 and 1580 cm^−1^ approx. which correspond to the D and G bands respectively [33]. In carbon aerogels, the D band can be associated with alternating ring vibrations in condensed benzene rings [34], while the G band can be associated with the development of the sp^2^ carbon structure throughout the material during the carbonization process. It should be noted that carbon gels are normally amorphous carbon materials. Besides this, the intensity of the G band (*I*_G_) with respect to its D band (*I*_D_) is higher in the Ni doped aerogels than in the case of Fe or Co samples, and among the Ni samples this ratio *I*_G_/*I*_D_ is clearly higher in ANi6 and ANi4 than in ANi1 (Table 2).

Table 2 collects the metal crystallite sizes estimated by applying the Scherrer equation, the mean particle sizes obtained from HRTEM, the *I*_G_/*I*_D_ ration obtained from Raman spectra, the chemical composition obtained by XPS and total metal content of the aerogels. Among the Ni samples, the mean nickel particle size clearly increases with the metal loading; however, the samples ANi6, ACo6 and AFe6 show a very similar value around 21 nm.

**Figure 8:**
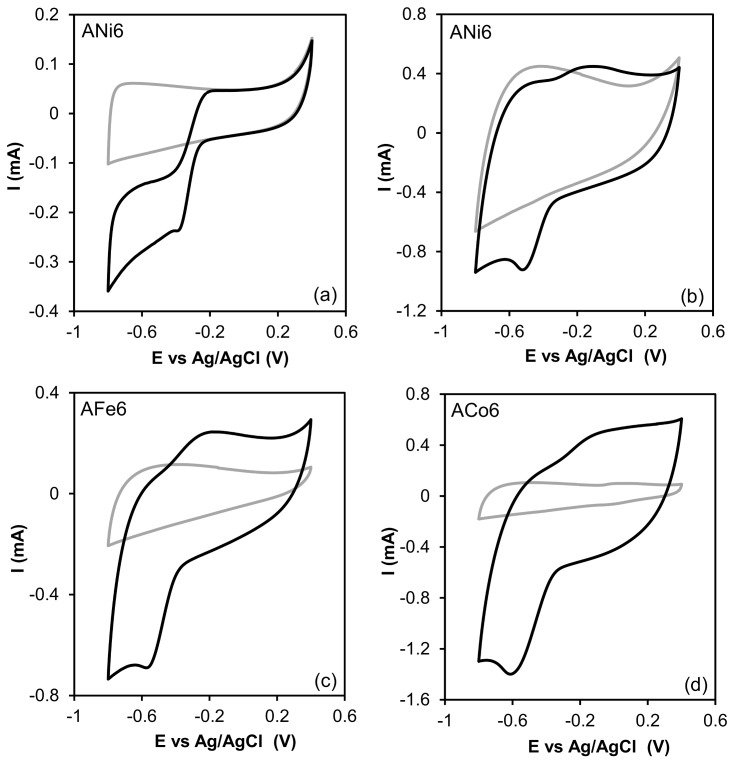
Cyclic voltammetries on N_2_-saturated KOH 0.1 M (grey) and O_2_-saturated KOH 0.1 M (black). (**a**) 5 mV·s^−1^; (**b**–**d**) 50 mV·s^−1^.

Regarding the Rotating Disk Electrode (RDE) experiments, cyclic voltammetry was used in order to observe the difference between the samples behavior on a N_2_-saturated electrolyte (KOH 0.1 M) and an O_2_-saturated one. Figure 8 shows CV curves for ANi6 sample at 5 mV·s^−1^ and at 50 mV·s^−1^, as well as for AFe6 and ACo6 samples at 50 mV·s^−1^ for comparison. In all cases a peak corresponding to the oxygen reduction can be observed when the curve is obtained on the O_2_-saturated electrolyte.

**Figure 9:**
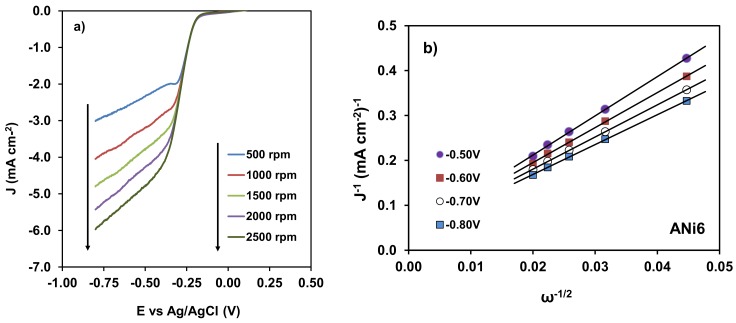
(**a**) LSV for ANi6 at different RDE rotating speed. (**b**) Koutecky-Levich fits at different potentials: from −0.5 to −0.8 V.

After CV, the electro-catalytic performance of the samples for oxygen reduction was studied by Linear Sweep Voltammetry (LSV). The experiments were conducted at a different rotating speed to apply the Koutecky-Levich Equation. This analysis is shown in Figure 9 for the ANi6 sample.

**Figure 10:**
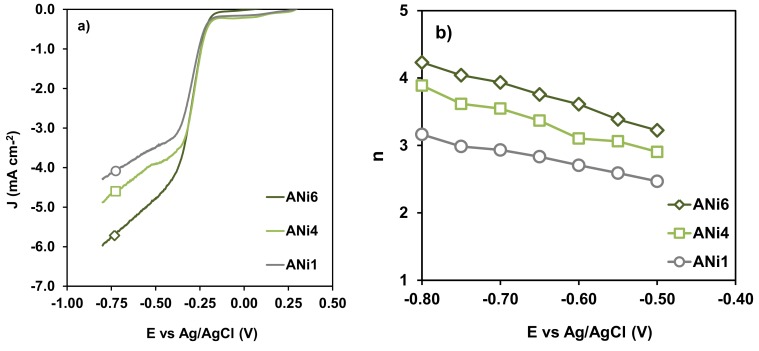
(**a**) LSV curves at 2500 rpm, and (**b**) variation of *n* with E vs. Ag/AgCl for samples ANi1 (◯), ANi4 (□), ANi6 (◇).

**Figure 11:**
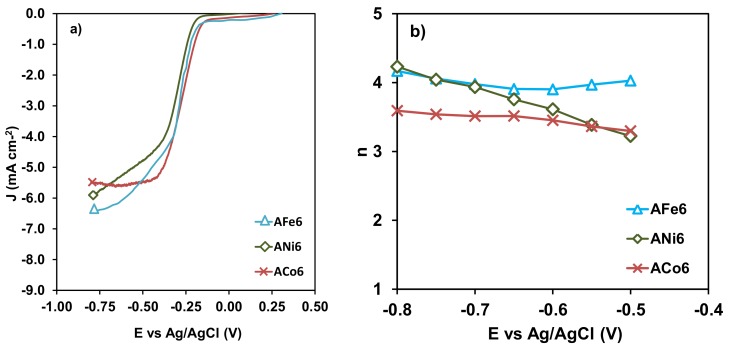
(**a**) LSV curves at 2500 rpm, and (**b**) variation of n with E vs. Ag/AgCl for samples AFe6 (Δ), ACo6 (×) and ANi6 (◇).

From this analysis the number of electrons transferred at a given potential can be obtained (Table 3). Aerogels with different content in Ni were tested to analyze the influence of the metal content in the electro-catalytic behavior of the samples on LSV (Figure 10a) and the number of electrons transferred (Figure 10b). Finally, aerogels doped with the three different metals but with the same metal loading (ANi6, ACo6 and AFe6) were compared as well (Figure 11). None catalytic activity was detected with the un-doped aerogel A0, neither by CV nor LSV.

## 4. Discussion

After analyzing the textural data collected in Section 3, it is concluded that metal-doped aerogels contain a very well-developed porosity, especially with a significant mesoporosity (*V*_BJH_). Aerogels doped with Ni have the highest surface areas and pore volumes, specially the highest micropore volumes. The metal phases are homogenously distributed into the carbon matrix and well dispersed. Doped aerogels contain a wide range of sizes of metal nano-particles, most of them with a zero-oxidation state (those embedded in the carbon matrix), with the exception of sample AFe6, which shows a mixture of Fe (0) and Fe (III). On the other hand, very low percentages of metal particles are detected in the external non-porous surface area, which would be partially oxidized. The macro-structure of these aerogels is similar among the samples; however, a partial graphitization process around the metal particles has also been detected in the case of the three different metals. In this line carbon aerogels doped with Ni seem to have the smallest and the best-developed graphitic clusters since their *I*_G_/*I*_D_ values are the highest [35], which could be due to the fact that they have the smallest metal particles (Figure 4 and Table 2).

Regarding electro-catalytic experiments, it can be observed that increasing the Ni loading improved the electro-catalytic performance of the aerogel (Figure 10). In fact, when the Ni percentage was really small (ANi1), the oxygen reduction reaction occurs through a combination of the 2 and 4 $e_{s}^{-}$, denoted by a value of *n* = 3.1 (Table 3). Nevertheless, as the Ni content increased, the number of electrons transferred also did, and the oxygen reduction occurred with an electronic transfer of 4 e^−^_s_ on both ANi4 and ANi6, although the reaction started at similar potentials as denoted by the value of *E*_onset_ (Table 3).

With respect to the type of metal, ACo6 is the best electro-catalyst showing the highest values of current density and the lowest value of *E*_onset_ among all the samples studied. On the other hand, some differences are also observed between ANi6 and AFe6 (Figure 11): ANi6 is the sample where the oxygen reduction occurs with a larger current density, although keeping the value of *E*_onset_ similar for both. According to bibliography [13,36,37], Ni-based electro-catalytic are in general less active than those-based in Fe or Co, this would be related to the ability of the metal to produce the dissociation of the oxygen molecule. However, in our material series, ANi6 show a very good electro-catalytic performance, even better than that for AFe6. In this case, ANi6 needs to be considered for its larger micropore volume and its smaller size of graphitic clusters. In fact, the electro-catalytic behavior of the carbon materials on the Oxygen Reduction Reaction is closely related to the type of carbon structure present in the material [38,39], and to its porosity. As was mentioned in previous paragraphs, the graphitic clusters for ANi6 are much smaller than those in the case of AFe6 and ACo6, therefore its good electro-catalytic performance could very well be related to it. In any case, it should be remarked that the catalytic results obtained with Ni-doped aerogels are especially interesting and much more in comparison to those obtained with Co- and Fe-doped ones.

On the other hand, we have included in Table 4 some bibliographic results obtained in similar experimental conditions to ours, and using as electro-catalysts Pt, Ni, Co, and Fe supported on different carbon materials. Pt/Carbon catalysts with a 20 wt % of Pt loading are a typical reference electrode [40,41,42,43,44,45], some authors use carbon black [40,43,44,45] and others prefer graphitic carbons as support [41,42]; in any case, all the collected results with this type of reference catalyst show the lowest *E*_onset_ potentials, which is reasonable because Pt itself is a better catalyst than the others, but the reported *j_k_* values are lower or similar to ours. Despite that, our catalysts have much lower metal loadings and they do not contain platinum. Similar conclusions are obtained when carbon aerogels (prepared from melamine) [13,36], graphene oxide [46] or carbon nanotubes [44,45,47] were used as support of Ni, Co, or Fe; therefore, our *j_k_* values are really good in comparison with those collected in Table 4. It is also remarkable that we have not found in the literature Ni-carbon-based electro-catalysts with a better performance than our ANi6 using similar ORR experimental conditions; its *j_k_* = 28.1 is really significant. Thus, some electro-catalysts with low metal contents show n-values close to 2 which are lower than those obtained with our electro-catalysts ANi1 (*n* = 3.1). Finally, Ni and Co unsupported nanoparticles [48] have been described as not catalytically active in this experimental ORR condition. These results, together with the fact that our un-doped A0 aerogel neither was active in the reaction, would indicate some type of catalytic synergism effect between the carbon and metal phases, especially taking in account that the accessibility of the metal particles to the electrolyte could be limited by the graphitic cluster developed around them.

Therefore, the results of this work clearly show that carbon aerogels doped with transition metals (obtained by polymerization of resorcinol and formaldehyde) are very good candidates as oxygen reduction electro-catalysts, where the current densities depend on the type and amount of metal doping and where the role of the carbon phase, both its textural and chemical properties, have a strong influence on the whole catalytic behavior of the material.

## 5. Conclusions

All the metal-doped carbon aerogels showed promising behavior in the oxygen reduction reaction; their well-developed porosity together with a very good metal dispersion in the carbon matrix, lead to materials with a very high electro-catalytic activity in ORR. As the Ni content was increased, the electro-catalytic behavior improved. Co-doped aerogel is the best electro-catalyst, showing the highest values of current density and the lowest value of E_onset_ among all the studied samples. Nevertheless, the nickel-doped aerogel (ANi6) presented even better results than the Fe one, which can be very well related to changes in the carbon crystalline structure and porosity, since ANi6 is the aerogel with the largest micropore and mesopore volumes and also the one with the smallest graphitic clusters. In general, the presence of small and well-developed graphitic domains seems to improve the electro-catalytic reduction of oxygen.

## Figures and Tables

**Table 1:** Name, surface areas and pore volumes of the doped carbon gels.

Sample	*S*_BET_	*W*_0_ (N_2_)	*L*_0_ (N_2_)	*W*_0_ (CO_2_)	*L*_0_ (CO_2_)	*V*_0.95_ (N_2_)	*V*_BJH_ (N_2_)	*L*_BJH_
	m^2^∙g^−1^	cm^3^∙g^−1^	nm	cm^3^∙g^−1^	nm	cm^3^∙g^−1^	cm^3^∙g^−1^	nm
A0	700	0.276	1.20	0.249	1.06	1.21	0.89	19.8
ANi1	663	0.258	1.07	0.276	0.63	0.82	0.54	17.1
ANi4	685	0.268	0.96	0.280	0.63	0.71	0.46	16.9
ANi6	698	0.273	0.90	0.294	0.64	0.69	0.48	16.8
ACo6	589	0.230	1.00	0.181	0.57	0.65	0.40	14.1
AFe6	461	0.177	1.00	0.182	0.62	0.41	0.25	12.3

**Table 2:** Chemical characteristics of the carbon aerogels.

Sample	Metal_TOTAL_	Metal_XPS_	*O*_XPS_	*d*_XRD_	*d*_HRTEM_	*I*_G_/*I*_D_
	wt %	wt %	wt %	nm	nm	
A0	n.d.	n.d.	1.4	n.d.	n.d.	-
ANi1	1.2	n.d	1.6	15.9	11.9	0.97
ANi4	3.9	n.d	1.6	17.4	15.5	0.99
ANi6	5.8	0.3	1.8	21.1	17.7	0.99
ACo6	5.9	0.7	3.6	21.5	19.4	0.92
AFe6	6.1	0.4	2.9	21.6	18.6	0.89

n.d: no detected.

**Table 3:** Parameters obtained from the analysis of LSV curves (values of *n* refer to K-L fitting for data at −0.8 V).

Sample	E_onset_	*j_k_*	*n*
	V	mA·cm^−2^	
ANi1	−0.22	16.6	3.1
ANi4	−0.21	16.9	3.9
ANi6	−0.21	28.1	4.2
ACo6	−0.17	34.9	3.6
AFe6	−0.22	26.2	4.1

**Table 4:** Comparison of our electro-catalysts with others found in the literature using similar conditions and electrolyte KOH 0.1 M.

Catalyst Name	Type of Support	*E*_onset_ vs. Ag/AgCl (V)	*n*	Ref.	Metal wt %	*j_k_* mA·cm^−2^
ANi6	Carbon aerogel	−0.210	4.2	This work	5.8	28.1
ACo6	Carbon aerogel	−0.170	3.6	This work	5.8	34.9
AFe6	Carbon aerogel	−0.220	4.1	This work	6.1	26.2
20% Pt Vulcan	Carbon black	−0.037	3.9	[40]	20	N.R.
20% Pt/C	Graphitic carbon	−0.050	3.9	[41]	20	5
20% Pt/C	Graphitic carbon	−0.070	4.2	[42]	20	28.8
20% Pt/C	Carbon black	−0.065	4.0	[43]	20	14
20% Pt/C	Carbon black	-	3.9	[44]	20	≈29 *
Pt/Vulcan	Carbon black	−0.007	3.9	[45]	20	N.R.
NT_FePc_400	Carbon nanotube	−0.037	3.9	[45]	2.1	N.R.
NT_CoPc_400	Carbon nanotube	−0.150	2.4	[45]	2.1	N.R.
Co-NCA	Carbon aerogel	−0.150	4.0	[36]	3	≈25 *
Fe-NCA	Carbon aerogel	−0.150	3.8	[36]	5.2	≈14 *
Fe-NCA5	Carbon aerogel	−0.051	3.8	[13]	7.7	≈25 *
FeCo-N-rGO	Carbon nanotube	0.050	3.9	[44]	0.46	≈25 *
CoNPs/rGO	Graphene oxide	−0.115	3.9	[46]	0.3	N.R.
Fe3C-CNTFs	Carbon nanotube	0.105	3.1	[47]	N.R.	4.89
Co-CNTFs	Carbon nanotube	−0.015	3.9	[47]	N.R.	5.23
Ni-CNTs	Carbon nanotube	0.055	2.6	[47]	N.R.	3.67
Ni	Unsupported	0	0	[48]	100	0
Co	Unsupported	0	0	[48]	100	0

(N.R.) *j_k_* values or *j*^−1^ vs. *ω*^−1/2^ plots not reported; (*) Data estimated from the corresponding *j*^−1^ vs. *ω*^−1/2^ plots.
